# Application on perioperative ERAS concept in elderly lung cancer patients undergoing surgery

**DOI:** 10.1097/MD.0000000000036929

**Published:** 2024-02-09

**Authors:** Ming Zhang, Ping Cai

**Affiliations:** a Department of Thoracic Surgery, Affiliated Hospital of Jiangnan University, Wuxi, Jiangsu, China.

**Keywords:** elders, enhanced recovery after surgery (ERAS), lung cancer, perioperative period

## Abstract

Investigating the applying effects of the enhanced recovery after surgery (ERAS) in the perioperative period of elderly lung cancer patients undergoing the surgery. We randomly selected 98 elderly patients with lung cancer who were admitted to our hospital and underwent surgery from January 2022 to September 2023 as study subjects. The control group received conventional care during the perioperative period, and the intervention group received ERAS-guided care measures. The differences in perioperative-related indices, pulmonary function, pain level, inflammatory factors, and postoperative complication rates between these 2 groups were compared. The postoperative extubation time, the activity time since getting out of bad and hospital stay were lower in the observation group than those in the control group (*P* *<* .05). At 3 days postoperatively, the FEV1, forced vital capacity and maximum ventilation volume of these 2 groups were lower than those of their same groups before surgery, and those of the observation group were higher than those of the control group (*P* *<* .05). At 3 days postoperatively, the numerical rating scale in both groups were lower than those of their same groups at 6 hours postoperatively, and the numerical rating scale of the observation group was lower than that of the control group (*P* *<* .05). At 3 days postoperatively, tumor necrosis factor-α, IL-6, and CRP in both groups were higher than those in their same groups before surgery, and those of the observation group was lower than those of the control group (*P* *<* .05). The incidence of postoperative complications in the observation group was lower than that in the control group (*P* *<* .05). ERAS applied in the perioperative period of elderly lung cancer patients undergoing surgery can shorten the hospital stay, promote the postoperative recovery on pulmonary function, alleviate inflammation, and reduce the risk of postoperative complications.

## 1. Introduction

Lung cancer is one of the most common malignancies clinically. It has shown an increasing trend in incidence and mortality in recent years, and a higher proportion of older patients compared with that of younger patients. In an epidemiological analysis of nonsmall cell lung cancer in the United States from 2010 to 2017 published in 2021,^[1]^ 67% of the 1.28 million new cases of nonsmall cell lung cancer patients were over 65 years old. Surgical treatment such as the radical resection on lung cancer is currently an effective treatment widely used in the clinical therapeutics of lung cancer patients. However, the treatment outcome is not only related to the lung cancer itself and the surgery. The physiological and psychological conditions of elderly lung cancer patients are significantly different from those of younger patients. Due to the low cardiopulmonary endurance, the falling physiological function and the various underlying diseases. Moreover, there is a large loss on postoperative pulmonary function, resulting in a relatively high incidence of postoperative complications.^[2]^ Systematic and effective nursing interventions in the perioperative period can play an important role in improving patients’ pulmonary function^[3,4]^ and reducing surgical stress and postoperative complications.^[4]^

Enhanced recovery after surgery (ERAS) is a concept that applies treatment and care measures guided by the ERAS concept in the perioperative treatment and nursing of patients to reduce the incidence of postoperative complications and alleviate the stress response in surgical patients, thereby promoting rapid recovery.^[5]^ Perioperative care is an integral part of the ERAS concept and is a concrete manifestation of patient orientation. As a new theory, a series of measures related on ERAS have been widely used in gastrointestinal surgery, cardiac surgery, and other fields, which have obvious benefits in reducing intraoperative and postoperative complications,^[6–8]^ as well as physical and psychological stress of patients and promoting functional recovery.^[9]^ For example, a META analysis of the effectiveness of ERAS in liver surgery showed^[10]^ that the ERAS group had a 2.22 days reduction in hospital stay (MD = –2.22; CI, –2.77 to –1.68; *P* *<* .001) and lower hospital costs (SMD = –0.98; CI, –1.37 to –0.58; *P* *<* .001) and a reduced risk of complications (RR, 0.71; 95% CI, 0.65–0.77; *P* *<* .001) compared with those of the standard treatment group. However, the application of the concept of care guided by ERAS in thoracic surgery has a relatively slow development, and the most of thoracic clinical departments are still using traditional care methods, which also limits the development of ERAS techniques. Based on this, the study objective of this research was to investigate the effect of ERAS in the perioperative period of elderly lung cancer patients undergoing surgery.

## 2. Method

### 2.1. Study subjects

This study, designed as a retrospective case-control investigation, received ethical approval from the Ethics Committee at Jiangnan University Affiliated Hospital. In this single-center retrospective analysis, we meticulously curated a cohort of 98 elderly patients diagnosed with lung cancer who were hospitalized and underwent surgical interventions at our institution from January 2022 to September 2023. Among these, 49 patients had previously received ERAS-conceptualized nursing care at our institution, while the remaining 49 patients received standard care protocols during their hospitalization. Inclusion criteria were as follows: (1) adherence to established clinical diagnostic criteria for lung cancer^[11]^; (2) enrollment of patients aged 60 years or older; (3) utilization of a surgical approach involving thoracoscopic lung lobe resection in conjunction with mediastinal lymph node dissection; (4) comprehensive preoperative assessments ensuring the absence of surgical or anesthetic contraindications and the medical endorsement for surgery; (5) a documented absence of prior history related to radiotherapy, chemotherapy, or antecedent malignancies. Exclusion criteria were defined as follows: (1) intraoperative deviations from the originally planned surgical approach, such as transitions from thoracoscopic to open thoracotomy procedures; (2) a documented history of severe trauma or prior thoracic surgeries; (3) the presence of concomitant severe cardiopulmonary dysfunction, lung infections, or hepatic and renal insufficiency; and (4) the co-occurrence of autoimmune diseases. Informed consent was diligently obtained from all patients and their respective families, with every procedural aspect of this research conducted in strict accordance with the principles delineated in the Helsinki Declaration.

### 2.2. Nursing method

ERAS-guided nursing measures were implemented on the basis of routine care.

1. Preoperative care: The content includes admission education, preoperative nutritional assessment, and preoperative airway management. ① This included medical history inquiries, orientation to the ward environment, surgical knowledge dissemination, risk and precautionary information, ERAS concept introduction, consent form signing, and patient and family member engagement to alleviate anxiety and fear. ② Preoperative patients are given nutritional risk assessment in a timely manner, and the assessment is done by using Nutritional Risk Screening-2002, which determines the nutritional status of patients according to the score. Giving targeted nutritional support according to the results. ③ Airway management: The physician assesses the patient preoperatively and conducts the necessary tests. For patients with airway risks, measures such as breathing training, the use of breathing trainers, stair climbing, effective coughing, and nebulized inhalation are taken before and after surgery for preventing and reducing airway problems.
2. Intraoperative care: ① Intraoperative temperature management: Controlling the room temperature in the operating room at about 23°C, and patients with hypothermia can use a thermal blanket to maintain their body temperature to ensure that the patient’s body temperature remains constant. The sodium chloride solution for flushing the thoracic cavity will be heated to 35–40°C, and infusion warmer making the temperature that should be maintained at 37.5°C for intravenous infusion. ② Intraoperative anesthesia: Employed a combination of general and epidural anesthesia, with precise control of anesthetic drug dosages to expedite postoperative extubation.
3. Postoperative care: ① The early mobilization on the postoperative stage: On the day of surgery, assisting patients to turn over and do upper and lower limb exercises; On the first day of surgery, in addition to continuing the exercises of the previous day, increasing the bedside sitting and standing training; on the second day of surgery, continuing the training on the day of surgery and carrying out the bedside walking training; on the third day of surgery, encouraging patients to get out of bed and do some exercises. ② Postoperative diet principles: Reasonable and balanced, fresh and varied, small but frequent. Patients can drink water 6 hours after waking up from anesthesia, and can eat liquid food when 12 hours passing by. At the next day after surgery, lunch and dinner can be semiliquid food, the light-flavor would be the best, adding high-quality protein appropriately. Egg custard, fine noodles, the minced meat and vegetable porridge, and so on are all acceptable, and the patient can eat normally 48 hours after surgery. The patient’s postoperative diet was given based on the guidance offered by the nutrition nurse in the department. ③ Postoperative pain management: Using intravenous self-control analgesic pump, offering paravertebral nerve block, prophylactically offering opioid and NSAID combination analgesic, and reducing opioid dosage. ④ Postoperative tube care: Paying attention on fixing the tube and reserving enough length to facilitate the patient’s movement; paying attention on checking whether the drainage tube is unobstructed, observing the color, nature and amount of drainage-fluid to avoid pressure and blockage; changing the drainage bottle regularly to prevent infection. At the same time, chest X-ray testing was carried out for the patient in order to remove the patient’s chest drainage tube as soon as possible and reduce the patient’s pain.

The control group mainly received routine care including preoperative admission education, nutritional support (stop eating 12 hours before surgery, give a small amount of food 12 hours after surgery), and postoperative analgesia (give opioids and nonsteroidal antiinflammatory drugs for pain relief). Strengthen intraoperative medical cooperation and observe patients’ vital signs in real time. The patient was given routine infusion without heating. Routine general anesthesia, intraoperative routine fluid rehydration.

### 2.3. Evaluation indicators

(1) Perioperative-related indices: The differences in the activity time since getting out of bed, the time of postoperative extubation, and hospital stay between these 2 groups of patients were counted and compared. (2) Pulmonary function: Patients were tested for pulmonary function before and 3 days after surgery, using a uniform pulmonary function instrument, including forced expiratory volume in 1 second (FEV1), forced vital capacity (FVC), and maximum ventilation volume (MVV). (3) Pain: At 6 hours and 3 days postoperatively, the pain level was assessed by using Numerical Rating Scale (NRS),^[12]^ with a NRS of 0–10, with higher scores indicating more intense pain. (4) Inflammatory factors: Serum inflammatory factor levels: 4 mL of venous blood was drawn in the early morning on an empty stomach before surgery and on the third postoperative day, and the serum was collected after centrifugation, and the levels of inflammatory factors, including tumor necrosis factor (TNF)-α, interleukin (IL)-6, and C-reactive protein (CRP), were measured by radioimmunoassay. (5) Incidence of postoperative complications: Complications such as pulmonary atelectasis, lung infection, poor incision healing, and respiratory failure that occurred after surgery were counted and compared between these 2 groups.

### 2.4. Statistical analysis

Data analysis was performed by using SPSS 23.0 software. The continuous variables were tested for normality and those confirmed to be normally distributed were expressed as X ± SD, and the statistical significance of differences in continuous variables between these 2 groups and within the groups at 2 time points was assessed by using independent sample *t*-tests and paired sample *t*-tests. Categorical variables were expressed as percentages and a chi-square test was performed, and *P* *<* .05 was considered as a statistically significant difference.

## 3. Results

### 3.1. Baseline data characteristics

During the study period, 98 participants who met the eligibility criteria were recruited, with 49 participants in each of the ERAS group and the control group. All patients in ERAS group had good compliance with ERAS protocol. There were no statistically significant differences between the observation group and the control group when comparing baseline information such as age, gender, body mass index, underlying disease, and type of pathology (*P* > .05). See Table 1.

**Table 1:** Comparison of baseline data characteristics among 2 groups [n (%), X ± SD].

Projects	Observation group (n = 49)	Control group (n = 49)	*t/X²*	*P*
Age (y)	66.08 ± 4.72	66.31 ± 4.92	0.230	0.818
**Gender**			0.400	0.527
Male	33 (67.35%)	30 (61.22%)		
Female	16 (32.65%)	19 (38.78%)		
BMI (kg/m²)	23.25 ± 3.72	22.97 ± 3.33	0.392	0.696
**Underlying diseases**				
Diabetes	25 (51.02%)	23 (46.94%)	0.163	0.686
Coronary heart disease	20 (40.82%)	24 (48.98%)	0.659	0.416
High blood pressure	35 (71.43%)	32 (65.31%)	0.424	0.514
Duration of disease (y)	1.47 ± 0.52	1.54 ± 0.54	0.618	0.537
**Type of pathology**			0.675	0.411
Adenocarcinoma	27 (55.10%)	31 (63.27%)		
Squamous carcinoma	22 (44.90%)	18 (36.73%)		

### 3.2. Comparison of perioperative-related indicators

The time of postoperative extubation, the activity time since getting out of bed and hospital stay were lower in the observation group than those in the control group (*P* *<* .05). See Table 2.

**Table 2:** Comparison of perioperative-related indicators in 2 groups (X ± SD).

Group	n	Postoperative extubation time (d)	Activity time since getting out of bed (h)	Hospital stay (d)
Observation group	49	0.83 ± 0.33	16.69 ± 1.67	3.16 ± 0.37
Control group	49	1.02 ± 0.40	20.09 ± 1.78	3.53 ± 0.50
*t*		4.129	9.743	4.098
*P*		<0.001	<0.001	<0.001

### 3.3. Comparison of pulmonary function

**Figure 1.**
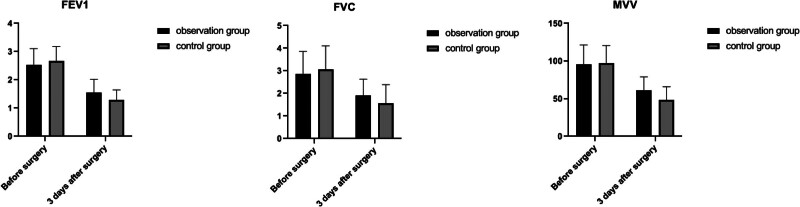
Comparison of pulmonary function indices in 2 groups.

Before surgery, the FEV1, FVC, and MVV of these 2 groups were compared, and the differences were not statistically significant (*P* > .05); 3 days after surgery, the FEV1, FVC, and MVV of these 2 groups were lower than those of their same groups before surgery, and those in the observation group was higher than those in the control group (*P* *<* .05). See Table 3 and Figure 1.

**Table 3:** Comparison of pulmonary function in 2 groups (X ± SD).

Projects	Observation group (n = 49)	Control group (n = 49)	*t*	*P*
FEV1 (L)				
Preoperative	2.36 ± 0.39	2.41 ± 0.29	1.116	0.267
Postoperative 3	1.58 ± 0.23Δ	1.31 ± 0.27Δ	3.161	0.002
FVC (L)				
Preoperative	2.89 ± 0.99	3.01 ± 1.06	0.873	0.384
Postoperative 3 d	1.90 ± 0.71Δ	1.60 ± 0.84Δ	2.372	0.019
MVV (L/min)				
Preoperative	87.20 ± 10.07	82.20 ± 10.86	0.259	0.796
Postoperative 3 d	58.66 ± 6.23	49.31 ± 7.61	3.507	<0.001

Compared with the situation before surgery,

Δ *P* *<* .05.

FEV1 = forced expiratory volume in 1 second, FVC = forced vital capacity, MVV = maximum ventilation volume.

### 3.4. Comparison of pain scores

**Figure 2.**
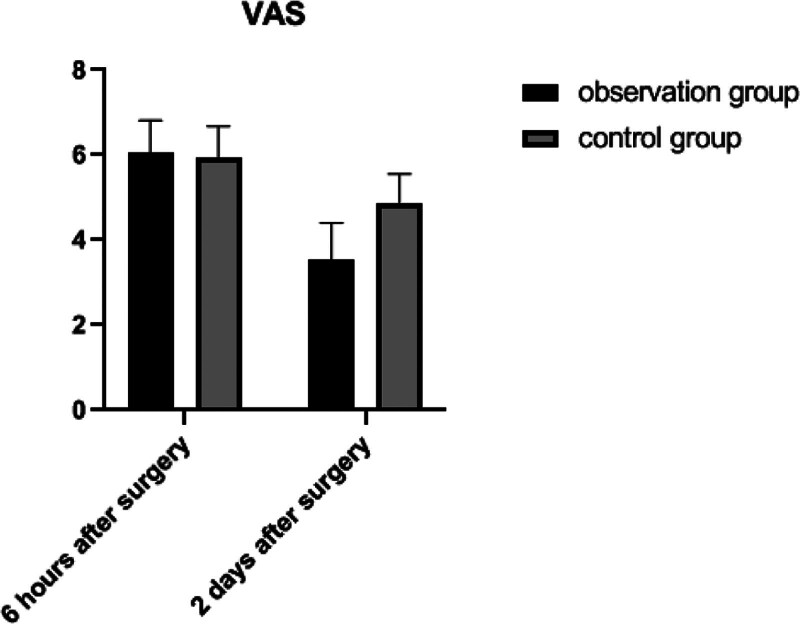
Comparison of NRS in 2 groups. NRS = Numerical Rating Scale.

At 6 hours postoperatively, the NRS of these 2 groups were compared, and the difference was not statistically significant (*P* > .05); At 2 days postoperatively, the NRS of both groups were lower than those of their same groups at 6 hours postoperatively, and the NRS in the observation group was lower than that in the control group (*P* *<* .05). See Table 4 and Figure 2.

**Table 4:** Comparison of NRS in 2 groups (X ± SD).

Group	n	6 h postoperative	2 d after surgery	*t*	*P*
Observation group	49	6.06 ± 0.75	3.54 ± 0.86	15.542	<0.001
Control group	49	5.95 ± 0.73	4.88 ± 0.67	8.389	<0.001
*t*		0.769	8.556		
*P*		0.444	<0.001		

### 3.5. Comparison of inflammatory factors

**Figure 3.**
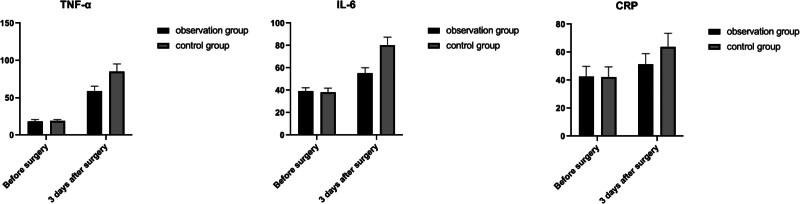
Comparison of inflammatory factors in 2 groups.

Preoperatively, the TNF-α, IL-6, and CRP of these 2 groups were compared, and the differences were not statistically significant (*P* > .05); 3 days after surgery, the TNF-α, IL-6, and CRP of these 2 groups were higher than those of their same groups before surgery, and those in the observation group was lower than those in the control group (*P* *<* .05). See Table 5 and Figure 3.

**Table 5:** Comparison of pulmonary inflammatory factors in 2 groups (X ± SD).

Projects	Observation group (n = 49)	Control group (n = 49)	*t*	*P*
TNF-α (μg/L)				
Preoperative	18.67 ± 2.14	18.79 ± 2.06	0.268	0.789
Postoperative 3 d	59.17 ± 6.25Δ	85.40 ± 9.96Δ	15.602	<0.001
IL-6 (pg/mL)				
Preoperative	39.03 ± 3.25	38.15 ± 3.72	1.241	0.217
Postoperative 3 d	55.13 ± 5.03Δ	80.38 ± 7.06Δ	20.379	<0.001
CRP (pg/mL)				
Preoperative	42.77 ± 7.11	42.34 ± 7.25	0.301	0.763
Postoperative 3 d	51.33 ± 7.68Δ	63.96 ± 9.52Δ	7.220	<0.001

Compared with the situation before surgery,

Δ *P* < .05.

### 3.6. Comparison on the incidence of complications

The incidence of postoperative complications in the observation group was lower than that in the control group (*P* *<* .05). See Table 6.

**Table 6:** Comparison of postoperative complication rates in 2 groups [n (%)].

Projects	Observation group (n = 49)	Control group (n = 49)	*X²*	*P*
Pulmonary atelectasis	2 (4.08%)	4 (8.16%)		
Lung infection	1 (2.04%)	3 (6.12%)		
Poor incision healing	1 (2.04%)	4 (8.16%)		
Respiratory failure	1 (2.04%)	2 (4.08%)		
Total incidence	5 (10.20%)	13 (26.53%)	4.355	0.036

## 4. Discussion

Lung cancer is a prevalent malignant tumor, which is mostly treated by surgery in clinic. Conventional lung tumor resection procedures tend to be more invasive and associated with a higher incidence of postoperative complications.^[13]^ Endoscopic surgery has fewer postoperative complications compared with traditional surgery due to its smaller trauma to patients. However, postoperative pain and stress reactions on patients are still unavoidable. Therefore, there are higher requirements for patients’ disease observation and care in the perioperative period. ERAS concept is a multidisciplinary collaboration of anesthesia, minimally invasive, and interventions on helping patients’ recover as soon as possible, and it has gradually developed into a perfect systematic and efficient interventional approach.^[14,15]^ The ERAS pathway has been applied by many surgical specialties, and rapid perioperative recovery aims to improve postoperative recovery through evidence-based practices, including early activity, multimodal analgesic medications, nutritional optimization, and the alleviation of patient psychological stress. The choice of surgical type has a significant impact on postoperative recovery. The observational studies of patients undergoing lung cancer surgery have shown that thoracoscopic lobectomy has a better prognosis compared with open thoracotomy, it is less traumatic with the lower stress reactions form patients, and contributes to rapid postoperative recovery.^[16]^ Moreover, the thoracoscopic lung cancer radical resection can reduce pain, improve shoulder function, enhance the early activity without the reliance of bed, shorten the hospital stay, better maintain pulmonary function, and improve quality of life, and prolong higher 5-year survival.^[17]^

The ERAS concept facilitates postoperative recovery by reducing postoperative complications in patients, which in turn reduces hospital stay. Another study of Martin et al^[18]^ was conducted on ERAS protocols for thoracoscopic lobectomy and thoracic surgery. It was concluded that ERAS reduced the hospital stay of patients by 2 days. Finally, a recent prospective study by Rogers et al,^[19]^ who examined a univariate analysis of overall adherence on the ERAS pathway and the 15 separate components of the pathway showed that early mobilization and carbohydrate loading adherence were significantly associated with reduced mortality and shorter hospital stay. Our results showed that the mean hospital stay in the observation group was 3.16 ± 0.37 days, postoperative extubation time was 0.85 ± 0.36 days, and the activity time since getting out of bed was 16.69 ± 1.67 hours. However, the mean hospital stay in the control group was 3.53 ± 0.50 days, postoperative extubation time was 1.18 ± 0.42 days, and the activity time since getting out of bed was 20.09 ± 1.78. The postoperative extubation time, the activity time since getting out of bed and hospital stay in the observation group were lower than those in the control group (*P* *<* .05). This may be related to the patients’ preoperative pulmonary function training, early postoperative oral feeding, reduction of intravenous infusion, alleviation of patients’ pain, reduction of postoperative complications, and acceleration of patients’ recovery, which in turn reduces hospital stay.^[20]^

A pulmonary function test is a measurement of lung’s volume, capacity, flow rate, and gas exchange that shows how well the lungs are functioning. It is used to diagnose and determine the treatment of certain lung diseases. Pulmonary function is a useful indicator to monitor and assess a patient’s recovery after surgery and is one of the key concerns of perioperative thoracic surgeons. ERAS has been shown to be effective in thoracic surgery in improving postoperative alveolar dilatation and reducing the incidence of pulmonary complications. For example, the results of a previous study showed that patients in the ERAS group had an effective improvement in postoperative pulmonary function and facilitated the recovery of exercise capacity compared with those in the patients from control group.^[21]^ In our study, it has shown that FEV1, FVC, and MVV were lower in both groups at 3 days postoperatively than in those in their same groups preoperatively and were those in the observation group were higher than those in the control group (*P* *<* .05). This suggests that ERAS-guided nursing measures can effectively protect patients’ pulmonary function and promote the postoperative recovery of pulmonary function. This may be related to the preoperative teaching of respiratory function exercises and the use of respiratory function trainers. The preoperative respiratory function exercises aims to train patients’ respiratory movements, improve their coughing efficiency, instruct them on postural drainage or other ways to promote sputum elimination, improve their respiratory efficiency, and reduce the accumulation of intrapulmonary secretions.^[22,23]^ In addition, after surgery, ERAS-guided early activities, including turning, bedside activities, and the activities apart from the area of bed, those can lead to increased respiratory amplitude, enhanced effective gas exchange, easier discharge of secretions from the airways, reduced incisional infections, atelectasis, and the incidence of pleural effusion, promote the recruitment maneuver on lungs, and improve patients’ pulmonary function.^[24]^

Postoperative pain can lead to labored cough, restricted activity, emotional distress, and decreased immunity, and is a major factor affecting the recovery and postoperative hospital stay of patients with lung cancer. Actively reducing pain caused by surgery and optimizing drainage tube management can help patients on their cough and sputum excretion after surgery, maintain airway patency, enhance lung ventilation, and reduce the incidence of pulmonary infections and respiratory-related complications such as atelectasis. At the same time, it helps patients to leave bed early after surgery, improve blood circulation, prevent complications such as the deep vein thrombosis on lower limb and even pulmonary embolism, and improve physical function and quality of life. Therefore, perioperative analgesia is very important for patients’ rapid postoperative recovery. While the traditional concept of postoperative analgesia is to administer the drug when the patient feels pain, ERAS takes a multimodal approach on analgesia with regular and timed administration. The use of multimodal analgesia significantly improves analgesia and reduces the postoperatively systemic opioid consumption and reduces the short-term side effects of opioid including nausea, vomiting, intestinal obstruction, urinary retention, delirium, and drowsiness.^[5,25]^ With the increasing efficacy of multimodal analgesic regimes, opioids are progressively being used only for the rescue analgesia and not as the primary postoperative regime. High adherence to nonopioid enhanced recovery regimes has significantly reduced opioid requirements and improved outcomes. Huang et al^[26]^ conducted a study of ERAS combined with thoracoscopic surgery for lung cancer showing that NRS were lower in the ERAS group than those in the control group on the postoperative day 3. Our results showed that at 2 days postoperatively, NRS were lower in both groups than those in their same groups at 6 hours postoperatively, and NRS in the observation group were lower than those in the control group (*P* *<* .05).

CRP is an acute phase protein synthesized by hepatocytes when the body is exposed to inflammatory stimuli (e.g., infection or tissue injury) and is part of the nonspecific immune machinery. All types of surgery cause varying degrees of inflammation and injury, and CRP is elevated during the first few hours of inflammation production, peaks at 48 hours, and decreases to normal levels as tissues, structures and functions recovered, objectively reflecting the stress response and degree of injury to the body from surgery. In addition, IL-6 and TNF-α are important inflammatory mediators in the body,^[27]^ IL-6 can induce inflammatory response in the body, thus increasing the differentiation level of lymphocytes and enhancing the inflammatory response, and TNF-α can induce the production of acute proteins, improve the proliferation capacity of macrophages, and enhance the immune capacity of the body. Factors such as surgical trauma, postoperative pain, and postoperative infection may enhance the inflammatory response of patients, so postoperative measures on accelerating recovery, relieving pain, and reducing the risk of infection on patients can improve the level of inflammatory factors in patients. The results of this study showed that the TNF-α, IL-6, and CRP of these 2 groups were higher than those of the preoperative levels in their same group, 3 days after surgery. Those in the observation group were lower than those in the control group (*P* *<* .05). It indicates that the implementation of ERAS concept can reduce the postoperative inflammatory response in patients undergoing the radical resection on lung cancer. For the possible reason, under the guidance of ERAS, patients in the observation group started to do the activity since getting out of bed on the postoperative day accompanied by medical and nursing staff, and then gradually extending the activity time, which could reduce the incidence of related complications and the risk of infection.^[28]^ In addition, under the guidance of ERAS, a combination of analgesic pump, nonsteroidal antiinflammatory drugs, and local infiltration anesthesia was used to relieve patients’ pain and reduce the inflammatory response.^[29,30]^

The core of ERAS is to reduce trauma and stress reactions and to reflect the safety of the treatment process. Of particular importance is the prevention of postoperative complications and the incidence of symptoms that affect quality of life. Madani et al^[31]^ reported 234 patients and found that the implementation of the ERAS protocol reduced the incidence of total complications from 50 to 37% in the ERAS group, with a significant decrease in the rate of urinary tract infections. The results of this study indicate that the postoperative complication rate in the observation group was lower than that in the control group (*P* *<* .05). It is suggested that ERAS can reduce the risk of postoperative complications in lung cancer patients. The reason is that the attraction and application of the ERAS concept in perioperative care provided optimal and scientific nursing interventions to patients, reducing trauma and promoting postoperative recovery.

There are limitations in this study. First, the small sample size included in the study and the fact that the study subject was only recruited from our institution may lead to biased results. Second, the observation follow-up period of this study was short, and the observation follow-up period was ended only until the patients were discharged from the hospital, and there was no follow-up to observe the effect on the subsequent changes in pulmonary function over a longer period of time. This suggests that we need to expand the sample size in the next study and refine the study design for further investigating the results of this study.

In summary, ERAS applied in the perioperative period for elderly lung cancer patients undergoing surgery can shorten the hospital stay, promote the postoperative recovery on pulmonary function, and reduce inflammation and the risk of postoperative complications.

## Author contributions

**Conceptualization:** Ming Zhang, Ping Cai.

**Data curation:** Ming Zhang.

**Formal analysis:** Ming Zhang.

**Investigation:** Ming Zhang, Ping Cai.

**Methodology:** Ping Cai.

**Software:** Ping Cai.

**Supervision:** Ping Cai.

**Validation:** Ping Cai.

**Writing – original draft:** Ming Zhang, Ping Cai.

**Writing – review & editing:** Ming Zhang, Ping Cai.
